# High‐definition transcranial direct current stimulation (HD‐tDCS) of left dorsolateral prefrontal cortex affects performance in Balloon Analogue Risk Task (BART)

**DOI:** 10.1002/brb3.884

**Published:** 2018-01-11

**Authors:** Heng Guo, Zhuoran Zhang, Shu Da, Xiaotian Sheng, Xichao Zhang

**Affiliations:** ^1^ Beijing Key Laboratory of Applied Experimental Psychology National Demonstration Center for Experimental Psychology Education Faculty of Psychology Beijing Normal University Beijing China

**Keywords:** decision making, high‐definition transcranial direct current stimulation, left dorsolateral prefrontal cortex, risk‐taking

## Abstract

**Background:**

Studies on risk preferences have long been of great concern and have examined the neural basis underlying risk‐based decision making. However, studies using conventional transcranial direct current stimulation (tDCS) revealed that bilateral stimulation could change risk propensity with limited evidence of precisely focalized unilateral high‐definition transcranial direct current stimulation (HD‐tDCS). The aim of this experiment was to investigate the effect of HD‐tDCS focalizing the left dorsal lateral prefrontal cortex (DLPFC) on risk‐taking behavior during the Balloon Analogue Risk Task (BART).

**Methods:**

This study was designed as a between‐subject, single‐blind, sham‐controlled experiment. University students were randomly assigned to three groups: the anodal group (F3 anode, AF3, F1, F5, FC3 returned), the cathodal group (F3 cathodal, AF3, F1, F5, FC3 returned) and the sham group. Subsequently, 1.5‐mA 20‐min HD‐tDCS was applied during the BART, and the Positive Affect and Negative Affect Scale (PANAS), the Sensation Seeking Scale‐5 (SSS‐5), and the Behavioral Inhibition System and Behavioral Approach System scale (BIS/BAS) were measured as control variables.

**Results:**

The cathodal group earned less total money than the sham group, and no significant difference was observed between the anodal group and the sham group.

**Conclusions:**

These results showed that, to some extent, focalized unilateral cathodal HD‐tDCS on left DLPFC could change performance during risky tasks and diminish risky decision making. Further studies are needed to investigate the dose effect and electrode distribution of HD‐tDCS during risky tasks and examine synchronous brain activity to show the neural basis.

## 1 INTRODUCTION

Risk‐based decision making is an essential advanced cognitive function in daily life and has long been a concern of researchers in different fields, such as economics (Kahneman & Tversky, 2000), management (Sitkin & Weingart, 1995), and psychology (Gardner & Steinberg, 2005).With the development of cognitive neuroscience, a body of evidence for the neural correlates of risk‐taking has accumulated in recent years (Tom, Fox, Trepel, & Poldrack, 2007). Neuroimaging studies have shown that the dorsal lateral prefrontal cortex (DLPFC) is a vital region involved in neural networks of risk‐taking and has been associated with risky behaviors (Brevet‐Aeby, Brunelin, Iceta, Padovan, & Poulet, 2016; Rao, Korczykowski, Pluta, Hoang, & Detre, 2008; Steinberg, 2008). Risk‐taking process in BART mainly included conflict control (anterior cingulate cortex, ACC; Rao et al., 2008), value calculation and reward‐seeking (vmPFC; Fukunaga, Brown, & Bogg, 2012; Schonberg et al., 2012), self‐control and impulse control (dorsolateral prefrontal cortex, DLPFC; Schonberg et al., 2012), emotional information process (orbital frontal cortex, OFC; Hsu, Bhatt, Adolphs, Tranel, & Camerer, 2005), aversive somatic regulation (anterior insula; Rao et al., 2008), and so on. In this dynamic and reciprocal network, DLPFC played an important role in producing self‐control behavior and control the risky behavior. However, the shortcomings of neuroimaging research include a lack of causal relationship (Mandzia & Black, 2001; Miller & D'Esposito, 2005). Emerging brain stimulation techniques could provide compelling evidence for the causality, which to a certain degree, compensates for this limitation (Venkatakrishnan & Sandrini, 2012).

Typically, brain stimulation techniques involve invasive and noninvasive stimulation techniques (Fox et al., 2014). Noninvasive stimulation techniques are relatively safe for humans (Been, Ngo, Miller, & Fitzgerald, 2007), and in recent years, more studies have applied noninvasive stimulation, such as transcranial magnetic stimulation (TMS) and transcranial direct current stimulation (tDCS; Dayan, Censor, Buch, Sandrini, & Cohen, 2013). TMS uses a pulsed magnetic field to induce current flows in human brain, and thus the neural activity is affected (Hallett, 2000). Evidence from TMS research suggests that low‐frequency repetitive transcranial magnetic stimulation of the right DLPFC induces risk‐taking behavior (Knoch et al., 2006). Although TMS has an advantage in spatial resolution, transcranial direct current stimulation (tDCS), as a promising noninvasive method of brain stimulation that contains safe, portable, and inexpensive features, has been widely applied over the last decade (Gandiga, Hummel, & Cohen, 2006; Nitsche & Paulus, 2000; Poreisz, Boros, Antal, & Paulus, 2007). tDCS uses 1–2‐mA direct currents via electrodes to penetrate the brain and modulate the cortical excitability (Fregni & Pascualleone, 2007). TMS and tDCS are both neuromodulatory interventions, which can explore the causal relationship between brain and behavior (Filmer, Dux, & Mattingley, 2014). Compared to TMS, tDCS has an advantage in safety and cost, which has not any report of Serious Adverse Effect in humans (Bikson et al., 2016). Previous studies have reported that tDCS with the anode over the right and the cathode over the left DLPFC could diminish risk‐taking behavior (Fecteau, Knoch, et al., 2007). Furthermore, results that left anodal/right cathodal tDCS on DLPFC increases the choosing of risk items have been found in aged individuals (Boggio et al., 2010). Moreover, Fecteau, Pascual‐Leone, et al. (2007) found that both right anodal/left cathodal and left anodal/right cathodal DLPFC tDCS could lead to risk‐averse behavior in the Balloon Analogue Risk Task (BART). In contrast, unilateral DLPFC stimulation (anode left or anode right) shows no such effect. Based on these results, Weber, Messing, Rao, Detre, and Thompson‐Schill (2014) examined the neural effects of tDCS during BART and applied right anodal/left cathodal tDCS on the DLPFC. They found that the number of pumps was negatively related to the brain connectivity of the right DLPFC.

Taken together, these results suggest that compared with unilateral stimulation, bilateral frontal tDCS could reduce risk behavior. However, studies using transcranial alternating current stimulation (tACS) have demonstrated that left DLPFC stimulation enhances risk propensity in BART but that the propensity does not differ between the sham group and the right stimulation group (Sela, Kilim, & Lavidor, 2012). Without knowing the effect of modulation when using unilateral stimulation with different polarities, more evidence should be accumulated.

Conventional tDCS has a disadvantage of low spatial resolution because the stimulation position is usually based on a cortical region (Datta et al., 2009). Unilateral DLPFC conventional stimulation usually has a farther distance between the electrode montages than bilateral stimulation, resulting in a lower current density (Faria, Hallett, & Miranda, 2011). Thus, the experimental effect may be diminished. Recently, as a novel method, high‐definition transcranial direct current stimulation (HD‐tDCS) has been developed to compensate for the restrictions of conventional tDCS (Alam, Truong, Khadka, & Bikson, 2016). Having the advantage of high spatial resolution and focalizing, HD‐tDCS typically applies a 4 × 1 ring electrode configuration to focus the targeted cortical region more precisely compared with conventional tDCS (Hogeveen et al., 2016). Faria et al. (2011) noted that using four small return electrodes may be more effective than using the traditional large electrode. Magnetic resonance imaging (MRI)‐based finite element model (FEM) analysis has been used to simulate the current distribution (Datta, Truong, Minhas, Parra, & Bikson, 2014). By this method, researchers have compared the spatial resolution between conventional and HD‐tDCS and found that conventional tDCS had diffuse current flows, while the high‐definition ones had current flows constrained in the ring with radius about 3.5 cm (Kuo et al., 2013). Previous studies using HD‐tDCS have demonstrated the effects of modulating cortical excitability on working memory capacity (Tan, Ting, & Chan, 2015), using spatial navigation in healthy, aged individuals (Hampstead & Hartley, 2015), and improving treatment of aphasia (Richardson, Datta, Dmochowski, Parra, & Fridriksson, 2015). Nevertheless, there is scarce evidence on the application of HD‐tDCS to study advanced cognitive functions, such as risk‐based decision making. In addition, it remains unclear whether the precisely modulated activation of the left DLPFC affects risk‐based decision making. To partially compensate for this shortcoming, we used HD‐tDCS to regulate the left DLPFC in this study.

Based on previous studies, in this research, we attempted to investigate the effect of HD‐tDCS focalizing the left DLPFC on risk‐taking behavior during the BART. We hypothesized that anodal HD‐tDCS on the left DLPFC would increase risk‐taking behavior but that cathodal HD‐tDCS on the left DLPFC would diminish risk propensity according to previous studies. To our knowledge, this study is the first to apply unilateral HD‐tDCS to the risk‐taking task of BART, focusing on whether focalized unilateral HD‐tDCS would change risk‐based decision making. The beneficial behavioral effects of HD‐tDCS may have potential clinical and therapeutic applications (Richardson et al., 2015) for the treatment of risky problems and dysfunctions (Brunoni et al., 2012). The widely used BART was selected as a measurement to assess risk‐taking behavior (Lejuez et al., 2002). Furthermore, BART is associated with a variety of real‐life risk behaviors, such as adolescent smoking (Krishnan‐Sarin et al., 2007), risky sexual behavior (Lejuez, Simmons, Aklin, Daughters, & Dvir, 2004), alcohol consumption (Fernie, Cole, Goudie, & Field, 2010), and drug use (Hopko et al., 2006). To control individual differences and personalities, emotional state, impulsiveness, and sensation seeking were assessed as between‐subject control variables using the Positive Affect and Negative Affect Scale (PANAS), the Behavioral Inhibition System and Behavioral Approach System scale (BIS/BAS), and the Sensation Seeking Scale‐5 (SSS), respectively. In addition, for the safe consideration of subjects, the level of perceived pain was rated during the research.

## 2 METHODS

### 2.1 Participants

Fifty‐eight students (37 females and 21 males; mean age = 20.4 ±3.0 years) from Beijing Normal University participated in the study. All subjects were right‐handed and had no history of neurologic or psychiatric disorders and no history of seizure or neurologic trauma by oral self‐reports when they were recruited. All had normal or corrected‐to‐normal vision and no metal implants in the brain. No participant took stimulants or calming drugs prior to the experiment. All subjects were naive to tDCS and BART and provided written informed consent after being informed of the safety and possible risks of HD‐tDCS. The experimental design and procedures were approved by the Ethical Committee of Beijing Normal University.

### 2.2 Experimental design and procedure

**Figure 1:**
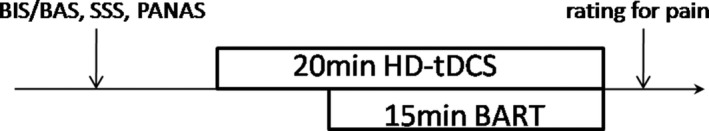
The experimental design

This study was designed as a between‐subject, single‐blind, sham‐controlled experiment. The participants were randomly assigned to three groups: the anodal group (*n* = 20, F3 anode, AF3, F1, F5, FC3 returned), the cathodal group (*n* = 16, F3 cathodal, AF3, F1, F5, FC3 returned), and sham group (*n* = 22). Prior to the study, participants were asked to complete the Chinese versions of PANAS (Watson, Clark, & Tellegen, 1988), BIS/BAS (Carver & White, 1994), and SSS (Zuckerman, 1979) to, respectively, measure emotion state, impulsiveness, and sensation seeking. After the premeasurement, HD‐tDCS was conducted for 5 min, and the BART began with the HD‐tDCS continuing. The total stimulation time was approximately 20 min, and the task was completed in approximately 15 min. Following the task, the subjects were asked to rate the degree of pain on a 7‐point scale. After completing the experiment, each participant was given some reward (Figure 1).

### 2.3 High‐definition transcranial direct current stimulation (HD‐tDCS)

**Figure 2:**
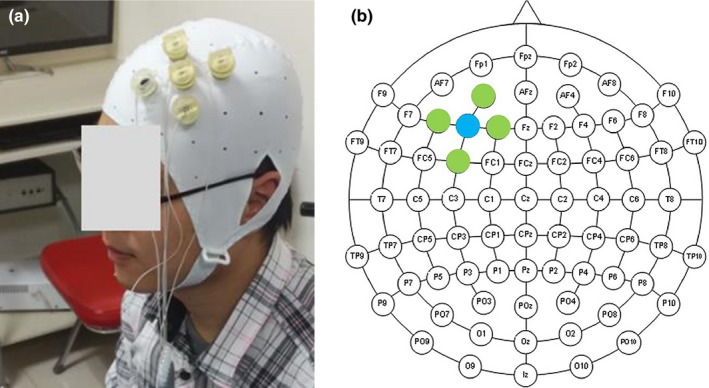
(a) A example applying HD‐tDCS in the study. (b) The center electrode was placed at F3 according to the International 10‐10 EEG System. The other four return electrodes were placed over AF3, F1, F5, and FC3

A battery‐driven, constant‐current DC‐stimulator and a distributor (4 × 1‐C3, Soterix Medical, New York, NY, USA) were used to deliver 1.5‐mA HD‐tDCS for approximately 20 min. Each of the five Ag/AgCl sintered ring electrodes had an approximately 4 cm^2^ connection to the scalp (Bo et al., 2016), generating a current density under the center electrode of approximately 3.7 A m^2^. To stimulate the left DLPFC, the center electrode was placed in F3 according to the International 10‐10 EEG System. The other four return electrodes were placed over AF3, F1, F5, and FC3 (Nikolin, Loo, Bai, Dokos, & Martin, 2015; see Figure 2). Prior to stimulation, the hair under the electrode was separated to expose the scalp, and approximately 2 ml of conductive gel (SignaGel; Parker Laboratories, Inc, Fairfield, NJ, USA) was placed into the electrode casings above the scalp. The polarity of the center electrode determined whether the stimulation was anodal or cathodal. For the actual stimulation, the current was applied for approximately 20 min (the exact length depended on the time that the subject completed the task), including an initial 30 s of ramping up and final 30 s of ramping down at the end. For the sham stimulation, the current was ramped up to 1.5 mA in 30 s and immediately ramped down to 0 in the following 30 s. This procedure was useful to blind the subjects to whether they received the sham stimulation (Garnett & Ouden, 2015). Previous studies have shown that 1.5‐mA HD‐tDCS is safe in healthy subjects (Gözenman & Berryhill, 2016). In our study, we asked the participants to rate the pain on a 7‐point scale ranging from 1 = no feeling to 7 = strongly pain after the stimulation. All participants tolerated the HD‐tDCS well, and no one reported side effects at one day after the end of the experiment.

### 2.4 Balloon analogue risk task (BART)

During the BART (Lejuez et al., 2002), the participants were asked to inflate 30 balloons by clicking the “pump” button on the computer screen using a mouse. They were told that, after the experiment, they would be rewarded with the final amount of money in the permanent bank. For each pump, the participants would receive one virtual coin stored in a temporary bank, and they could choose to continue pumping or terminate the inflation. After terminating, the money in the temporary bank would be transferred to the permanent bank. However, the balloon had a certain probability of explosion. If the balloon exploded, then the money stored in the temporary bank would be lost, and the participants would start to pump a new balloon. As the balloon became larger, the probability of explosion increased. The subjects were blinded to the explosion probability and were instructed to accumulate the largest amount of money in the permanent bank. The balloon has an average explosion point of 16. For each trial, the probability that the first pump would explode was 1/32, the second pump was 1/31, and so on. The 32nd pump’s exploding probability was 1/1. We calculated the earning, number of explosions, and adjusted number of pumps as dependent variables. The adjusted number of pumps referred to the average number of pumps for the unexploded balloons (Aklin, Lejuez, Zvolensky, Kahler, & Gwadz, 2005).

### 2.5 Self‐reports

#### 2.5.1 PANAS

Previous research has revealed that negative emotion could affect risk‐taking behavior (Suhr & Tsanadis, 2007). Therefore, the Chinese version of the Positive Affect and Negative Affect Scale (PANAS, Watson et al., 1988) was used to measure emotional state and consists of two dimensions: Positive Affect (PA) and Negative Affect (NA). Each dimension has 10 items. The participants were asked to respond on a 5‐point scale ranging from 1 = *never* to 5 = *always*. This scale has good psychometric properties (Serafini, Malinmayor, Nich, Hunkele, & Carroll, 2016).

#### 2.5.2 BIS/BAS

Evidence suggests that impulsivity could affect the BART (Lauriola, Panno, Levin, & Lejuez, 2014). In the present study, impulsivity was measured using the Chinese version of Behavioral Inhibition System and Behavioral Approach System scales (BIS/BAS, Carver & White, 1994), comprising 18 items measured on a four‐point scale from 1 = *strongly disagree* to 4 = *strongly agree*. The scale was divided into four subscales: Drive scale (BASD), Fun seeking scale (BASF), Reward responsiveness scale (BASR), and Behavioral inhibition scale (BIS). The Chinese version of BIS/BAS has satisfactory psychometric properties (Li, Zhang, Jiang, & Li, 2008).

#### 2.5.3 SSS

Studies have shown that sensation seeking is associated with the BART (Humphreys, Lee, & Tottenham, 2013). The Chinese version of the Sensation Seeking Scale‐5 (SSS‐5;Wang et al., 2000) was used to measure sensation seeking; it consists of 40 statements divided into four subscales: Thrill and Adventure Seeking (TAS), Experience Seeking (ES), Disinhibition (DIS), and Boredom Susceptibility (BS).The respondents were asked to answer *yes/no*, with the higher total score reflecting higher sensation seeking. This scale has good psychometric properties (Zhang, 1990).

### 2.6 Data analysis

The data were processed and analyzed using the software package SPSS 20.0. The primary outcome measurements were used as the BART values. To investigate the effect of Group (anodal/cathodal/sham), as a between‐participant independent variable, and Time (first 10 trials, mid 10 trials, last 10 trials), as a within‐participant independent variable (Fecteau et al., 2007), mixed‐design, repeated‐measure ANOVA was used to analyze scores on BART as dependent variables. The interaction effect of Time and Group was aimed to test whether the 5‐min warming up of stimulation was sufficient. If 5‐min prior stimulation were sufficient, there would be no difference between three groups over different time points, and the tDCS effect would be stable. In addition, the effect of emotion was assessed using PA and NA with a one‐way analysis of variance (anova) with Group as between‐subject factors. A one‐way anova was also used to compare the mean BIS/BAS and SSS scores of different groups. Bonferroni's correction was used for post hoc analysis. *p < *.05 was used as the statistical significance level. All results are presented as the mean ± *SD*.

Additionally, two alternative statistic methods (Permutation test and Bayesian analysis) were conducted additionally to test how large the difference was between groups (Kruschke, 2013), particularly the marginal significance. Permutation test and Bayesian analysis provided evidence to learn and assess the differences between groups, which avoided test H0 in Null Hypothesis Significance Testing (NHST; Kruschke, 2014). We conducted Bayesian estimation using JAGS 4.2.0 and R 3.4.0 following Kruschke's methods (Kruschke, 2013, 2014), which has been applied in HD‐tDCS study for statistical analysis (Hogeveen et al., 2016). We made region of practical equivalence (ROPE) between −1.0 and 1.0, and for more details, see Hogeveen et al. (2016). Nonparametric permutation test has been used in fMRI and ERPs statistical analysis for solving multiple comparison problems (Lage‐Castellanos, Martínez‐Montes, Hernández‐Cabrera, & Galán, 2010; Nichols & Holmes, 2002). We conducted permutation test by writing statements in R 3.4.0 for comparing critical group differences, following Butar & Parks’ basic steps (2008): First, calculated the mean difference “*D*_obs_” of the two groups and pooled the two groups into a new data set. Then, resampled two new groups from the data set and calculated the mean difference “*D*”. Third, repeated the step for 9999 times and drew the probability density curve of the mean differences. Therefore, we could decide where the *D*_obs_ was in this probability density curve.

## 3 RESULTS

All participants tolerated the HD‐tDCS well and completed the entire study. The pain rating revealed that the main effect of Group was not significant [*F*(2,55) = 0.43, *p *=* *.65, η^2^ = 0.015], indicating that the subjective pain level was similar among the three different groups. The side effects that the participants perceived included burning and itching sensations. For demographic data, chi‐square test was conducted for gender, and no difference was observed [χ^2^(2, *n *=* *58) = 1.28, *p *=* *.53]. Moreover, no significant effect of age was detected [*F*(2,55) = 2.19, *p *=* *.12, η^2^ = 0.074], suggesting that there was no difference in demographic composition between the groups.

For the self‐report data (Table 1), one‐way anova, with Group (anodal/ cathodal/ sham) as the independent variable, was performed to analyze the SSS. The result showed no significant effect of Group on the total score [*F*(2,55) = 0.78, *p *=* *.47, η^2 ^= 0.028]. Specifically, the main effect of Group was not significant on the four dimensions [TAS, *F*(2,55) = 0.97, *p *=* *.38, η^2 ^= 0.035; ES, *F*(2,55) = 0.71, *p *=* *.50, η^2 ^= 0.025; DIS, *F*(2,55) = 0.38, *p *=* *.68, η^2 ^= 0.014; BS, *F*(2,55) = 0.26, *p *=* *.77, η^2 ^= 0.009]. Furthermore, to analyze the Behavioral Inhibition System and Behavioral Approach System, we used one‐way anova with Group (anodal/ cathodal/ sham) as the independent variable and the BIS/BAS scores as dependent variables. There was no significant effect of Group in terms of BASR [*F*(2,55) = 0.29, *p *=* *.75, η^2 ^= 0.010], BASD [*F*(2,55) = 0.222, *p *=* *.80, η^2 ^= 0.008], BASF [*F*(2,55) = 0.95, *p *=* *.39, η^2 ^= 0.034], and BIS [*F*(2,55) = 2.06, *p *=* *.14, η^2 ^= 0.070]. Moreover, the total BIS/BAS score was not significant (*p *=* *.89). In addition, we investigated whether the groups differed in emotion state using one‐way anova, with Group (anodal/ cathodal/ sham) as the independent variable and the PANAS scores as dependent variables. In addition, no significant effects were found for PA [*F*(2,55) = 0.99, *p *=* *.38, η^2 ^= 0.035], or NA [*F*(2,55) = 0.77, *p *=* *.47, η^2 ^= 0.027]. In summary, the participants in different groups showed no significant differences in control variables containing emotional state and personality trait regarding impulsivity and sensation seeking.

**Table 1:** Descriptive statistics of the three groups are presented as follows (*M ± SD*)

	Anode (*n* = 20)	Cathodal (*n* = 16)	Sham (*n* = 22)	*F*	*p*
Age	21.30 ± 3.8	19.25 ± 0.9	20.41 ± 3.97	2.19	.12
Positive affect (PA)	15.55 ± 6.4	16.00 ± 6.4	18.14 ± 6.3	0.99	.38
Negative affect (NA)	27.70 ± 7.6	29.75 ± 7.6	26.95 ± 5.9	0.77	.47
BIS/BAS score	7.65 ± 1.0	7.80 ± 1.3	7.61 ± 1.5	0.12	.89
Drive scale (BASD)	1.94 ± 0.5	2.05 ± 0.4	2.00 ± 0.5	0.22	.80
Fun seeking scale (BASF)	1.95 ± 0.4	2.13 ± 0.4	2.08 ± 0.48	0.95	.39
Reward responsiveness scale (BASR)	1.66 ± 0.3	1.64 ± 0.4	1.73 ± 0.4	0.29	.75
Behavioral inhibition scale (BIS)	2.1 ± 0.4	1.99 ± 0.5	1.80 ± 0.5	2.06	.14
Sensation seeking scale‐5 score	21 ± 6.6	18.75 ± 5.7	19.41 ± 4.7	0.78	.47
Thrill and adventure seeking (TAS)	6.75 ± 2.0	5.63 ± 3.0	6.27 ± 2.3	0.97	.38
Experience seeking (ES)	5.45 ± 2.2	4.63 ± 2.0	5.05 ± 2.0	0.71	.50
Disinhibition (DIS)	4.65 ± 2.3	4.19 ± 2.0	4.14 ± 1.8	0.38	.68
Boredom susceptibility (BS)	4.15 ± 1.9	4.31 ± 1.4	3.95 ± 1.25	0.26	.77
Pain rating	2.30 ± 1.6	2.63 ± 1.0	2.59 ± 1.0	0.43	.65
Number of explosions	11.50 ± 4.06	11.44 ± 4.53	11.05 ± 4.63	0.07	.94
Earnings	180.70 ± 38.44	154.25 ± 34.40	187.64 ± 49.67	3.10	.05
Adjusted pumps	10.24 ± 3.08	8.93 ± 3.33	10.79 ± 4.51	1.16	.32

**Figure 3:**
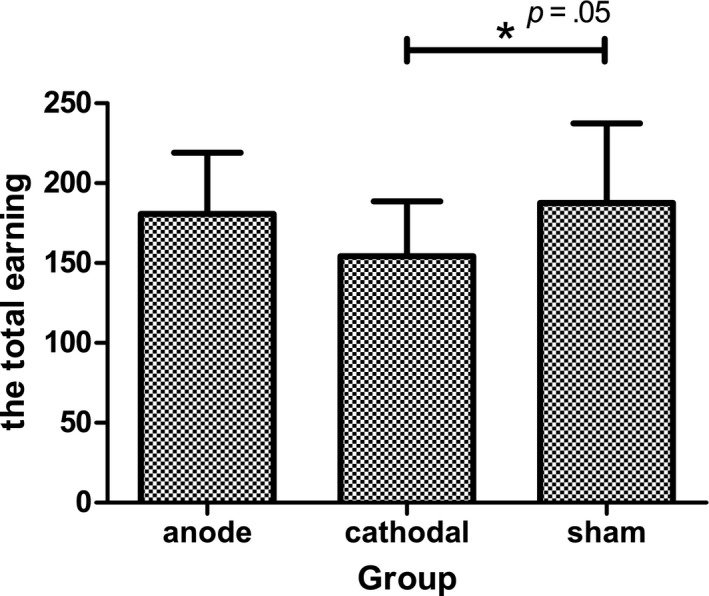
The total earnings for the three groups

**Figure 4:**
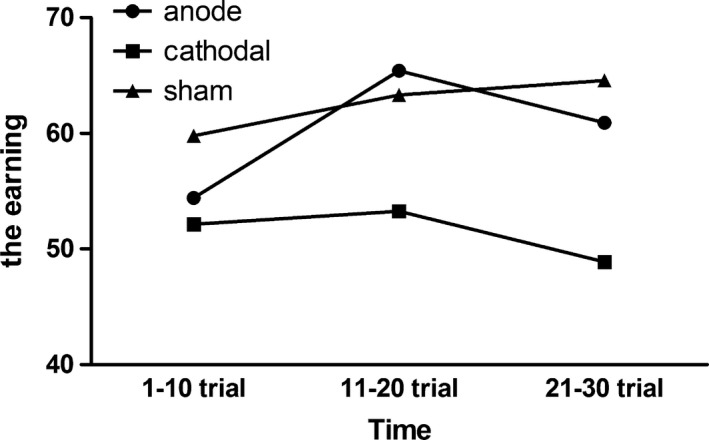
Mean earning for each of the 10 trials

The goal of our study was to investigate the effect of HD‐tDCS focalizing DLPFC on risk‐taking behavior during the BART. To this end, as the primary measurement, three indicators of BART were analyzed using repeated measures anova as dependent variables: the earning, the number of explosions, and the adjusted number of pumps. The independent variables were Group (anodal/ cathodal/ sham) and Time (first 10 trials, mid 10 trials, and last 10 trials). For the earnings (Figure 3), the anova revealed a main effect of Group [*F*(2,55) = 3.10, *p *=* *.05, η^2 ^= 0.101]. Furthermore, a marginal significance was detected using post hoc analysis. Compared to the sham group, the cathodal group earned less money (*p *=* *.058). In contrast, no effect was found when the cathodal group was compared with the anodal group (*p *=* *.2). These data indicated that participants who received cathodal stimulation earned less money than did those who received sham stimulation. Neither the main effect of Time [*F*(2,110) = 0.98, *p *=* *.38, η^2 ^= 0.018] nor the interaction between Group and Time was significant [*F*(4,110) = 0.50, *p *=* *.74, η^2 ^= 0.018] (Figure 4). Despite the interaction between Group and Time was not significant, we found a marginal significant for the earning of 21–30 trial between three groups (*p *=* *.06), while for the earning of 1–10 trial we found no significance (*p *=* *.58; See Table 2). This result implied that the difference of earnings between three groups became larger as the time went on.

**Table 2:** Descriptive statistics of the earning for each of 10 trials in three groups are presented as follows (*M ± SD*)

	Anode (*n* = 20)	Cathodal (*n* = 16)	Sham (*n* = 22)	*F*	*p*
1–10 trial	54.40 ± 28.32	52.13 ± 17.29	59.77 ± 22.09	0.55	.58
11–20 trial	65.40 ± 15.35	53.25 ± 21.52	63.32 ± 24.11	1.70	.19
21–30 trial	60.90 ± 24.49	48.88 ± 13.61	64.55 ± 19.98	2.91	.06

**Figure 5:**
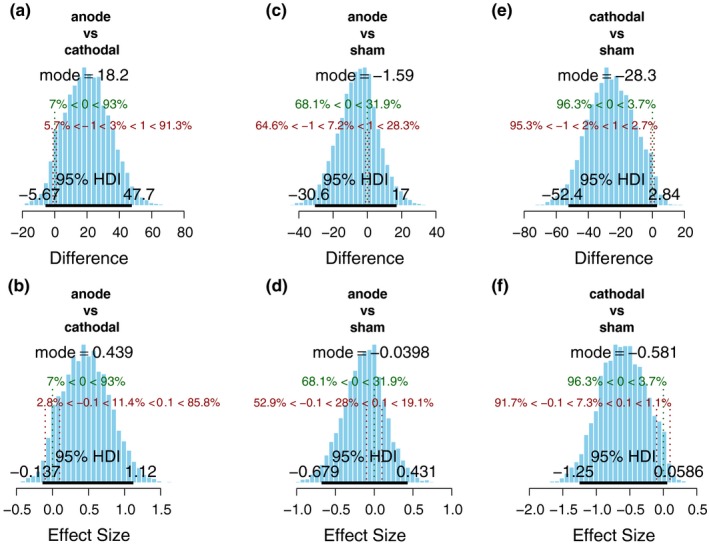
Bayesian estimation was conducted on earning to compare the difference between three groups

To compare the difference on earnings between three groups, Bayesian estimation was conducted. The results showed that the cathodal group was different from sham group (Mode = −28.3, 95% HDI = −52.4 to 2.84, Figure 5e). The effect size distribution had 7.3% in ROPE (Figure 5f). The cathodal group was also different from the anode group to some extent (Mode = 18.2, 95% HDI = −5.67 to 47.7, Figure 5a), and the effect size distribution had 11.4% in ROPE (Figure 5b). However, the anode group was equivalent to the sham (Mode = −1.59, 95% HDI = −30.6 to 17, Figure 5c), and the effect size distribution had 28% in ROPE (Figure 5d), revealing this two groups were almost the same.

**Figure 6:**
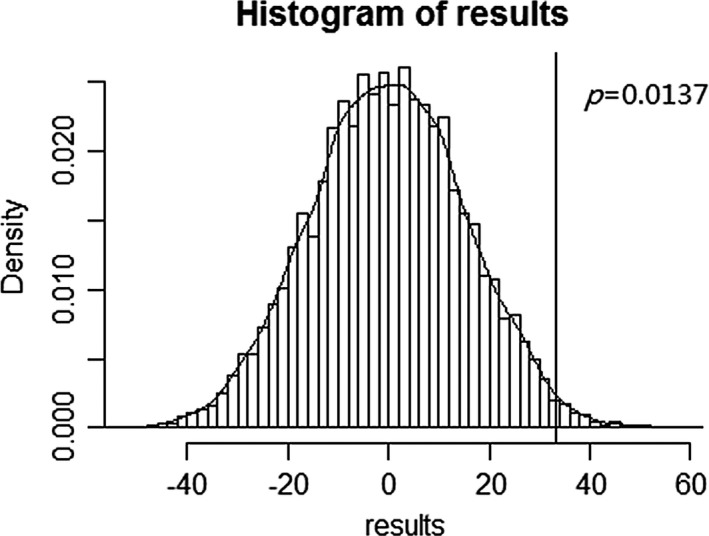
The result of permutation test on the difference between the cathodal and the sham group for total earnings. This probability distribution indicated the mean difference in resampling. The horizontal axis represented the mean value difference. The vertical line showed the mean difference in the actual sample

To verify the difference between the cathodal group and sham group on total earnings, we further performed a permutation test. The results revealed the mean difference in actual sample was higher than 98.63% mean value differences in the resampling (*p *=* *.0137, Figure 6), which provide evidence for confirming the difference between the cathodal group and sham group.

**Figure 7:**
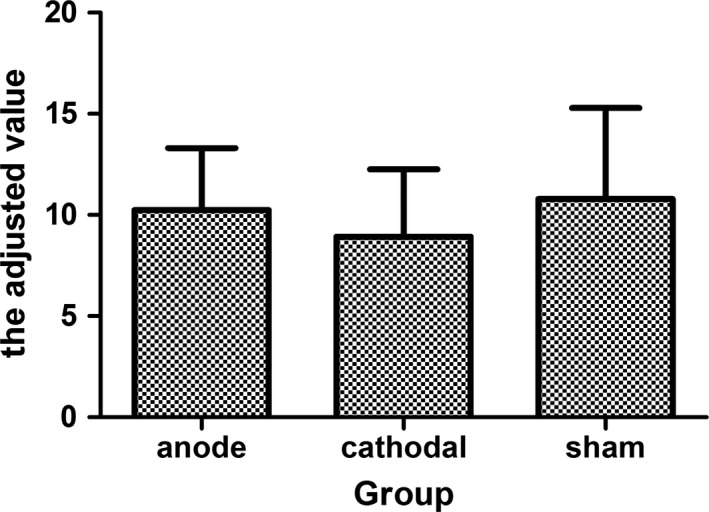
Mean‐adjusted value for each group

Furthermore, we investigated the effects of HD‐tDCS on the number of explosions and adjusted number of pumps (Figure 7). The results showed no significant effect of Group [*F*(2,55) = 0.07, *p *=* *.94, η^2 ^= 0.002], Time [*F*(2,110) = 0.13, *p *=* *.88, η^2 ^= 0.002], or the interaction between Group and Time [*F*(4,110) = 0.74, *p *=* *.57, η^2 ^= 0.026] on the number of explosions. For the adjusted number of pumps, anova revealed a nonsignificant effect for Group [*F*(2,55) = 1.16, *p *=* *.32, η^2 ^= 0.041], Time [*F*(2,110) = 1.96, *p *=* *.15, η^2 ^= 0.034], and the interaction between Group and Time [*F*(4,110) = 0.37, *p *=* *.83, η^2 ^= 0.013]. In addition, we conducted a correlation analysis between the adjusted number of pumps and the total earning and observed a significant correlation (*r *=* *.785, *p *<* *.001). In summary, the HD‐tDCS affected the index of the total earning, but not the adjusted number of pumps and the number of explosions.

## 4 DISCUSSION

Prior research has found that conventional bilateral tDCS on DLPFC could change risky behavior, but there is a lack of evidence for using precisely targeted HD‐tDCS focused on DLPFC. In the present study, we aimed to investigate the effect of HD‐tDCS focalizing the left DLPFC on risk‐taking behavior during the BART.

Self‐reports showed no significant differences between the three groups in terms of emotional state, sensation seeking, and impulsiveness. Evidence suggested that the three groups of participants were homogeneous in personality traits, consistent with the result of previous studies showing that emotion, sensation seeking, and impulsiveness could influence risky behavior during BART (Humphreys et al., 2013; Lauriola et al., 2014; Suhr & Tsanadis, 2007). In addition, the perceived pain did not differ between the groups, demonstrating that pain did not affect the BART results. Furthermore, our experiment supports the evidence that HD‐tDCS is tolerated and safe in health university students. The perceived pain value was lower than that reported in a previous study (Villamar et al., 2013), which is mainly due to the use of 1.5 mA stimulation intensity, compared with the 2 mA intensity used in the prior study.

The results of BART illustrated that participants receiving cathodal HD‐tDCS significantly earned less total money than the sham group. However, the interaction between Group and Time was not significant. We found a marginal significance for the earning of 21–30 trial between three groups (*p *=* *.06), and the simple effect was not so significant for 1–10 trial (*p *=* *.58; See Table 2). It implied that the tDCS effect may not be stable, as the group effect was largest in the last 10 trials. From the descriptive statistics, the difference between groups gradually increased over time. Therefore, the 5 min of prior stimulation should be lengthened in future study to make sure that the stimulation effect was produced completely at the beginning of task. The main effect of Time was not significant, which may suggest that practice effect and sequential effect did not have an impact on the experiment.

As the most widely used indicator, the adjusted number of pumps showed no significant difference. These results support the idea that unilateral HD‐tDCS on the left DLPFC is insufficient to change the adjusted number of pumps but is effective to influence the total money earned. Fecteau, Pascual‐Leone, et al. (2007) explained that the balance of activation across hemispheres hindered the effect of unilateral tDCS; to some extent, our study confirmed this view, showing that unilateral HD‐tDCS could not affect the adjusted number of pumps. However, unilateral cathodal HD‐tDCS produced behavioral effects. As the mean‐adjusted values of different groups were below the average explosion point, we propose that the total earning could also represent the risk preference, considering the high correlation between total earning and the adjusted value. Under the average explosion point, anticipated earnings increased with the number of inflations. As in Lejuez et al. (2002), the relationship between earned money and the number of pumps should be inverted “U” type. With highly risky behavior, the subjects would earn less money. In our study, the data showed all subjects’ mean‐adjusted pumps were 10.09 (*SD* = 3.76, Min = 4.17, Max = 19.38), which was lower than the average explosion point of 16. Therefore, in our data, with more pumps, the participants earned more money.

Although cathodal stimulation did not affect the common adjusted indicator, this stimulation technique evidently affected the task performance by making participants tend to be conservative, earning less money (Fecteau, Pascual‐Leone, et al. 2007), consistent with the results of previous studies(Fecteau, Knoch, et al., 2007). It is reasonable that precise and focalized unilateral HD‐tDCS could diminish risky behavior. Notably, these changes were confined to cathodal stimulation, and the anode showed no effect. Thus, the effect and strength of unilateral cathodal HD‐tDCS in the present study were not sufficiently high, and further studies are necessary to investigate the dose effect and electrode distribution.

To date, this study is the first to show the HD‐tDCS‐mediated effects on risky decision in BART, and evidence has been accumulated for focalized brain stimulation studies. These findings supported the argument that focalized unilateral cathodal HD‐tDCS on the left DLPFC could change performance in risky tasks and diminish risky decision making. In addition, anodal HD‐tDCS on the left DLPFC had no effects, consistent with previous studies. The present study may have certain clinical applications and can provide direction and reference for the treatment and intervention of impulsive disorders (Brunoni et al., 2012). Accumulating evidence for the application of tDCS in clinical samples, such as depression (Nitsche, Boggio, Fregni, & Pascual‐Leone, 2009), anxiety (Shiozawa et al., 2013), and meta‐analysis, revealed that tDCS is a promising technique for impulsive behavior therapy (Brevet‐Aeby et al., 2016). With a higher spatial resolution compared to conventional tDCS (Dayan et al., 2013), HD‐tDCS does not coactivate near brain regions and may have an increased therapeutic effect, which shows promise for improved therapeutic interventions in the near future.

HD‐tDCS has been used in surrounding brain areas near DLPFC. HD‐tDCS is considered a more focalized stimulation than conventional tDCS when targeted on motor cortex (Kuo et al., 2013). HD‐tDCS proved the casual relationship between the right lateral orbito frontal cortex and intention‐based cooperation (Zhang, Yu, Yin, & Zhou, 2016). Alam et al. (2016) investigated the spatial distribution of HD‐tDCS and found that, with wider stimulation radius, intensity at the center increased and spatial focality was lost. In our study, we chose a small stimulation radius, while the peak intensity was sacrificed. Chua, Ahmed, and Garcia (2017) used HD‐tDCS to separate the dissociable roles of prefrontal cortex and temporal lobe in memory tasks. For risk‐taking, future study may systematically compare the left and right frontal stimulation with different peak intensity and spatial resolution, which may help separate and understand the cortex function in risk‐taking.

There are several restrictions in the present study. One of the main limitations of the present study is the nonsignificance of the adjusted value, although the cathodal group had a lower adjusted number of pumps than did the sham group. This limitation likely reflects the lack of HD‐tDCS intensity and the length of prior stimulation. In conventional tDCS, 4‐mA stimulation has been considered (Nitsche & Bikson, 2017). For safe considerations, 1.5 mA was used in the present study; in future research, the dose effect and electrode distribution of HD‐tDCS require further clarification (Alam et al., 2016).

Furthermore, the left DLPFC has been associated with other functions, such as plan ability, working memory, and emotion regulation (Fecteau, Pascual‐Leone, et al. 2007). The activation of DLPFC involves modulating these advanced cognitive functions, which may be involved in the BART (Capone et al., 2016). Control tasks should be added in future to verify the stimulation indeed changed the risk‐taking behavior, rather than other cognitive functions (Maréchal, Cohn, Ugazio, & Ruff, 2017). For example, stimulating the left DLPFC could increase working memory capacity (Fregni et al., 2005), which was involved in the BART. Future studies will clarify and control the related variables and effects (Fecteau, Pascual‐Leone, et al. 2007).

There is increasing evidence supporting the argument that tDCS affects perception speed, attention, memory, and other relatively basic cognitive processing (Andrews, Hoy, Enticott, Daskalakis, & Fitzgerald, 2011; Fregni et al., 2005; Ohn et al., 2008; Zaehle, Sandmann, Thorne, Jancke, & Herrmann, 2011). However, only a few studies have applied tDCS for decision making or other advanced cognitive processing (Coffman, Clark, & Parasuraman, 2013). In the present study, BART was used as the risk task. Although BART has been associated with risky behavior in the real world, it cannot represent all risk scenarios. Therefore, further research is needed to use a wide variety of decision making tasks to test external validity (Pripfl, Neumann, Köhler, & Lamm, 2013).

Moreover, the neural mechanism by which HD‐tDCS affects risk‐based decision making is still not fully understood. Despite the growing body of studies using HD‐tDCS to examine behavioral effects, the neural mechanisms and processes of HD‐tDCS are currently unclear. The acquisition of brain activity signals concurrent with HD‐tDCS/ tDCS (Dayan et al., 2013), such as EEG‐tDCS (Cunillera, Brignani, Cucurell, Fuentemilla, & Miniussi, 2015; Roy, Baxter, & He, 2014), will help to uncover the “black box.”

In conclusion, in this study, we investigated the effect of HD‐tDCS focalizing the left DLPFC on risk‐taking behavior during the BART. We found that 1.5‐mA 20‐min HD‐tDCS could change risk‐taking behavior, suggesting that the left DLPFC is linked to risky propensity. Furthermore, this study has the advantage of high spatial resolution stimulation and appropriate control of between‐group differences. Our study possesses potential clinical and therapeutic applications for impulsive and risky behavior. However, the effect of HD‐tDCS is limited to a certain degree, and these results should be replicated and extended with larger current intensity. Follow‐up studies are encouraged to explore the dose effect and electrode distribution of HD‐tDCS during risky tasks. In addition, neuroimaging concurrent with HD‐tDCS will provide new insights into the neural basis of the tDCS effect.
